# Developing and integrating a destination decision support algorithm into an innovative electronic communication platform to improve injury care service coordination in Rwanda: The Rwanda912 study protocol

**DOI:** 10.21203/rs.3.rs-5640378/v1

**Published:** 2024-12-24

**Authors:** Rwanda RIGHT Group, Aurore Nishimwe, Justine Davies, Jean Claude Byiringiro, Abebe Bekele, Lucia D’Ambruoso, Agnieszka Ignatowicz, Barnabas Tobi Alayande, Jeanne D’Arc Nyinawankusi, Jean Marie Uwitonze, Jean Nepomuscene Sindikubwabo, Irene Bagahirwa, Collins Inkotanyi, Sudha Jayaraman, Antonio Belli, Rob Rickard, Assuman Nuhu, Ephrem Daniel Sheferaw, Laura Quiin, Gilbert Rukundo, Philbert Muhire, Oda Munyura, Fabien Hagenimana, Didier Hagumimana

**Affiliations:** University of Birmingham; University of Birmingham; University of Birmingham; University of Birmingham; University of Global Health Equity; University of Aberdeen; University of Birmingham; University of Global Health Equity; Rwanda Biomedical Center; Rwanda Biomedical Center; Rwanda Biomedical Center; Rwanda Biomedical Center; University of Global Health Equity; University of Utah; NIHR Surgical Reconstruction and Microbiology Research Centre; Rwanda Build Program; University of Rwanda; University of Global Health Equity; University of Birmingham; Rwanda Biomedical Center; Ruhengeri Level Two Teaching Hospital; University of Global Health Equity; University of Rwanda; University of Rwanda

**Keywords:** Emergency care, Pre-hospital emergency care system, Ambulance, Service d’Aide Medicale Urgente (SAMU), Destination Decision-Support Algorithm (DDSA), Rwanda

## Abstract

**Introduction:**

Delays in getting injured patients to hospital in a timely manner can increase avoidable death and disability. Like many low- or middle-income countries (LMICs), Rwanda experiences delays related to lack of efficient prehospital communication and formal guidelines to triage patients for hospital care. This paper describes the protocol to develop, roll out, and evaluate the effectiveness of a Destination Decision Support Algorithm (DDSA) integrated in an electronic communication platform, ‘912Rwanda’. The DDSA will facilitate the linkage of patients to health facilities able to treat their condition(s). Work will be conducted in three phases: i. development, ii. training and roll-out, and iii. evaluation.

**Methods and analysis:**

Work will be conducted in the pre-hospital emergency service “SAMU - Service d’Aide Medicale Urgente” and health facilities in Kigali City and Musanze District, which serve predominately urban and rural populations, respectively. We will develop interfaces to capture facility and patient-relevant data, which feed into a guideline-based electronic DDSA to match patients to hospitals. We will assess existing trauma care processes using qualitative and quantitative methodologies. This will be followed by a series of consensus workshops to develop at-scene triage guidelines and agree variables to capture in the interfaces. The DDSA will be developed based on outputs from these workshops and tested against historical ambulance data and expert opinion until acceptable thresholds of performance are passed. User interfaces will be developed and tested using human computer interface design principles.

**Discussion:**

The combined collaborative approach of bringing together experts and software developers, and with deep engagement of Rwandan stakeholders, including leadership of Rwanda Ministry of health through its technical arm, the Rwanda Biomedical Center, should lead to an ambulance communication system which is utilized, sustained, and effective.

## Introduction

Injuries are a substantial cause of death and disability [1]. They have a profoundly negative effect on both individuals and society [2]. Injuries cause about 4.4 million deaths globally, with tens of million more suffering from non-fatal injuries each year [1]. Adults of working age in low- and middle-income countries (LMICs) are primarily and disproportionately affected by injuries and trauma resulting in severe physical impairment, long-term disability, and psychological suffering [3].

A pillar of a good quality emergency care involves getting patients to the right health facility at the right time [4]. For severely injured patients, this should ideally be within one to two hours; a longer pre-hospital time is associated with increased mortality [5–7]. This is exemplified in patients with fractures in 18 LMICs, where 72% experienced delays for more than 2 hours before reaching hospital [8]. We have found that 40% of deaths after injury are avoidable and 40% of these were due to delays in getting to facilities [9–11]. The same holds for patients with time critical conditions [12].

In Rwanda, injury causes 9% of all deaths; 47% of these occur pre-hospital and 49% are within the first 24 hours of admission [13–15]. Road Traffic Incidents (RTIs) are a particular issue; in 2019 the Rwanda National Police documented 4,661 injuries and 700 fatalities owing to RTIs [16]. Of these, 35.6% had lifetime injuries, and around 50% had orthopedic issues [16]. As in many other LMICs, there are substantial delays in reaching treatment facilities in Rwanda [17–19].

In 2007, the Government of Rwanda through the Ministry of Health created a public ambulance service called the Service d’Aide Médicale d’Urgence (SAMU) [20]. This service was created to provide timely prehospital care, and to strengthen the health system [21]. SAMU has grown from a Kigali-based ambulance service to being a country-wide service covered by Community-based Health Insurance [21]. More than 300 ambulances are deployed across the country, linked by a national dispatch centre and a free emergency service number (912) [22]. SAMU transfers approximately 8,000 emergency patients per year, around 70% of whom have injuries [12], [23]. The remaining 30% have emergency medical or obstetric conditions [23]. Pre-hospital care services are provided by ambulance crews, and a data-based quality improvement programme was put in place to improve the quality of pre-hospital care [24].

**Figure 1:**
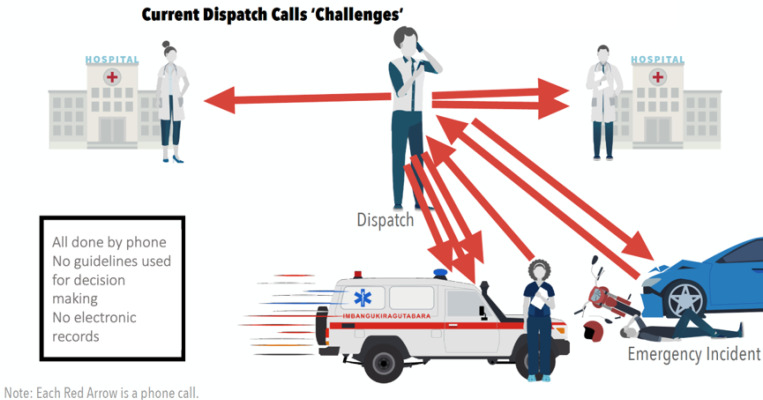
Inefficient communication (red arrows denote multiple mobile telephone calls) between dispatch centre, ambulance crews, and health facilities resulting in avoidable pre-hospital delays.

Similar to other ambulance services in African countries, all pre-hospital communications are done using mobile telephones [7]. We have identified that inefficient mobile telephone communication and coordination between ambulance, dispatch, and receiving hospitals (Figure 1), combined with a limited use of triage guidelines, results in an average journey time of 1h 15 minutes to reach hospitals in Kigali (unpublished data_Year 2023). This represents up to 30 minutes of additional time from the set SAMU target journey time (45 minutes) from emergency location to hospital, with 42% of trips taking longer than one hour (unpublished data).

Additionally, there are multiple potential health facilities to which emergency patients can be transported, however, ambulance crews have no formal guidelines to triage patients to the right facility for them. Although the reasons have not been formally assessed, 22% of all SAMU journeys are interfacility transfers (unpublished data), which potentially reflects a substantial number of patients for whom the initial facility selected didn’t have all the requirements to deal with their condition.

Reducing delays in patient care arising from inefficient pre-hospital communication requires intersectoral collaboration and in the setting, is best achieved through innovative, science-based, simple, low-cost, locally developed, and locally supported solutions. Building on formative work bringing together industry professionals, academics, and policy actors, 912Rwanda, a bespoke platform was developed by the Rwanda Build Program (RWBuild), a local software company, and implemented by SAMU in 2023. The goal of this project is to build on that platform and add the DDSA capabilities and further improve the efficiency of the pre-hospital care system by connecting patients to the nearest ready hospital to treat them. Appendix 1 describes the theory of change of this project.

### Aim

Our aim is to develop, roll out, and evaluate the effectiveness of a destination decision support algorithm (DDSA) added to the existing electronic communication platform ‘912Rwanda’ which will link patients requiring pre-hospital ambulance services to the closest facility that is ready and able to treat their condition(s). In this paper, we focus on the development, training, and roll-out (Study Objectives 1–3). The second element, evaluation (Objective 4) will be described in another sister paper.

## Methods

### Setting

Pre-hospital care and emergency services are being expanded in Rwanda and our descriptions represent the situation at the start of the project.

This work will be conducted in SAMU and health facilities in Kigali City and Musanze District in Rwanda. Kigali was selected for study given it is the largest conurbation in and capital city of Rwanda. Musanze district was selected as a rural site; although the district hosts the second largest city in Rwanda, the patient population catchment is predominantly rural.

In Kigali, there are five district hospitals (Kibagabaga, Masaka, Muhima, Nyarugenge, Kacyiru) and three referral hospitals (Centre Hospitalier Universitaire de Kigali, King Faisal Hospital, Rwanda Military Referral and Teaching Hospital. These hospitals receive emergency and trauma patients from the local area, many of whom are transported by SAMU ambulances [15]. The 36 health centres in Kigali, receive less urgent cases.

Ruhengeri Referral Hospital (RRH) is the main referral hospital in Musanze district. This hospital receives emergency patients from the local area, mostly via requests from health centres (primary health facilities), rather than from direct patient or bystander requests. Emergency patients are stabilized in RRH before being referred to Kigali if deemed necessary. Given the lack of ambulance services to transport patients from the scene of the emergency and that most emergency patients present initially to health centres, the identified need from our preliminary research is for a system to triage patient transfers from health centres to RRH. In Musanze there are usually five available ambulances; at any one time, two are generally occupied with transferring patients to Kigali. There is a dedicated team of seven nurses who oversee pre-hospital care.

### General description of 912Rwanda

The intervention, 912Rwanda, is designed and developed in two phases. In phase one which is now complete, the interfaces in dispatch centre, ambulances, and receiving health facilities and a software platform were developed, tested and implemented. Phase 1 developed the foundations of 912Rwanda, including a web-interface in dispatch to enter caller data, a map-based system to locate the patient, a mobile interface in ambulances to receive details of incidents and their location, and a process to send SMS messages to facilities to alert them as to incoming patients [25, 26]. This phase is funded by the National Institute of Health (NIH - 1R21TW011636–01A1) Fogarty Grant and an implementation report is in progress.

Phase two involves the development, testing, implementation and evaluation of the DDSA. This protocol describes phase two, which builds on the first phase [25, 26] and aims to develop a DDSA which utilizes information on facility and patient location (from phase one), the status of the patient (collected at the scene by SAMU staff), and the readiness of facilities to treat conditions (entered at health facilities).

#### DDSA inputs

**Figure 2:**
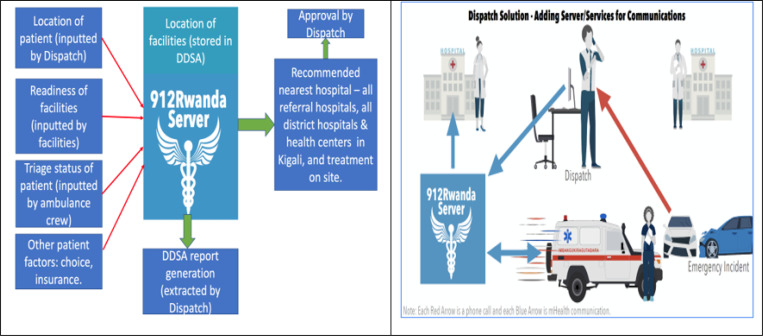
The Destination Decision Support Algorithm concept and solution

The DDSA will receive inputs from three main sources namely, ambulance, dispatch centre, and health facilities (Figure 2). The users of the system will enter routine data into interfaces. All data will be used for SAMU reporting and operations. A subset of these data will be fed through the DDSA as follows: dispatch enters details of the location of the emergency, the patient’s gender, and reported condition into a web interface in dispatch. This is sent to a mobile interface held by ambulance teams (this element has been developed, pilot tested, and rolled out in phase 1 [24]). At the scene, ambulance staff will input information on the clinical status of the patient and collect other patient-related factors relevant to the destination decision [27]. Dynamic facility readiness to receive patients (e.g. availability of staff, beds, equipment, and essential treatments [such as blood, anesthetics]) will be inputted by the facility staff at least twice daily. Additionally, static facility readiness (the usual readiness of facilities to treat different types of emergency patients) and the location of facilities is held in the DDSA.

The DDSA is expected to send an alert to the ambulance teams and/or dispatch of the selected facility and the rationale for this after receiving all inputs. This decision is either approved or overridden. If approved, the ambulance proceeds to the facility. If over-ridden, the ambulance team and/or dispatch manually enter their choice of facility and their rationale for this by means of a prepopulated survey form. This approval step as a human “check” on otherwise automated decisions, is standard in automated systems and is to mitigate against errors such as those seen in the introduction of fully automated ambulance software in London or the recent issues seen in the airline industry with Boeing 737 MAX [28, 29].

### Development, training and roll out research methodologies

**Figure 3:**
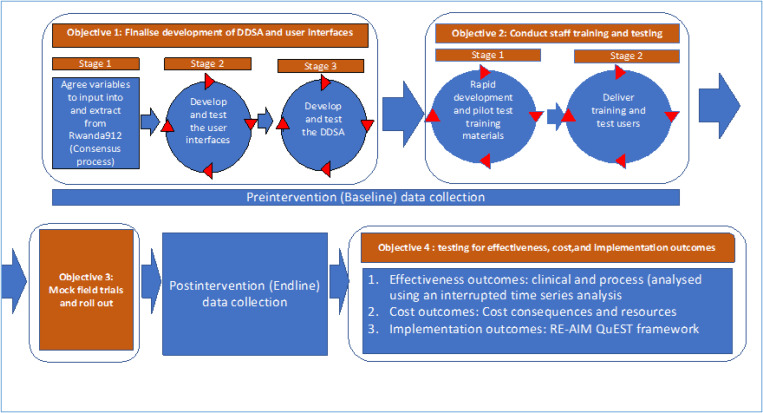
Summary of study flow

The flow of the three main stages (each represented as an objective) of the development phase is presented in Figure 3. Appendix 2 shows the proposed study timeline. Analyses of the processes will be done to maximize the potential to transfer results to other settings.

#### Objective 1: Develop 912Rwanda’s DDSA and user interfaces

**Figure 4:**
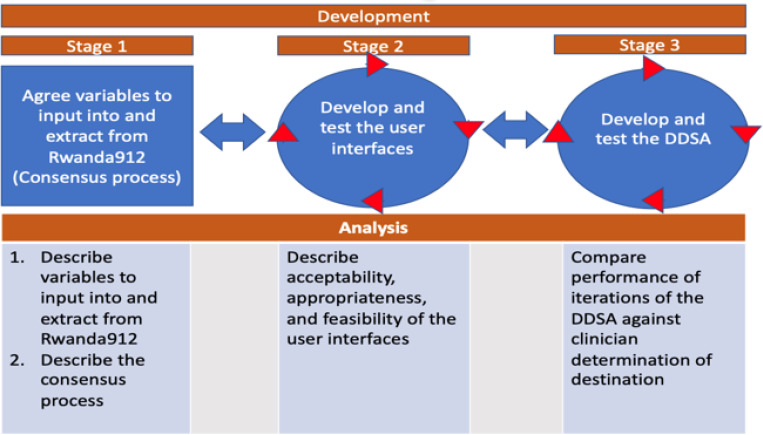
Objective 1 - stages and analysis

The first objective will be conducted in three stages: (1.1) to agree on variables to input into and extract from 912Rwanda system, (1.2) to develop and test the user interfaces, (1.3) to develop and test the DDSA (Figure 4). Objective 1 stages will be iterative, and there will be some overlap with potentially >1 stage being developed and tested at the same time.

To agree on variables to input into and extract from 912Rwanda, we will first appraise current data collection and use cases for emergency patients at SAMU (methods are described in Appendix 3). Findings will be presented at individual and multi-stakeholder workshops prior to discussions to gain consensus on variables which the DDSA will utilize.

User interfaces to input and receive information in ambulances, and facilities will be developed and tested using human computer interface (HCI) design principles through requirement analysis, designs and prototyping, as well as evaluation [30]. Based on findings from the ‘variable workshops’, and with input from experts in emergency medical data capture, user interfaces will be developed in consultation with the nominal lead of the relevant user group (SAMU ambulance staff, SAMU dispatch staff, facility staff, and MoH/RBC) and the research team leads. The interface prototypes will be iteratively developed until no further user improvement can be identified. (methods are described in Appendix 3)

The algorithms that form the DDSA have potentially varying degrees of complexity. An appropriate method will be chosen between decision trees (derived from basic manual or advanced-machine learning), Bayesian networks, or a hybrid approach (decision tree + Bayesian network). The configuration complexity chosen will depend on what is currently used for decision-making by SAMU and the amount of current data available to train and test an algorithm. Figure 2 illustrates the DDSA solution and concept. (methods are described in Appendix 3)

#### Objective 1 Analyses

Figure 4 describes the main analyses that will be done for each stage of the development in objective 1. In particular, the analysis for each stage will be done as follows:

##### Agreeing variables:

Priority lists and consensus outputs will be described using the terminology given by the group participants, with language adjusted when necessary for clarity. The records taken during the sessions will be used where clarity is needed in preparing the consensus outputs.

##### Meeting notes analysis:

Field notes on workshops proceedings will be analyzed qualitatively using thematic analysis with particular attention being given to the rapidity and challenges of developing consensus, participant interactions, facilitators and barriers to development of consensus, and the impacts of hierarchy on discussions.

##### User interfaces and DDSA testing:

User interfaces of each iteratively developed prototype will be tested using a collaborative heuristic approach [31] using three methodologies: scenarios and personas, prototype testing using HCI principles, and expert evaluation. Interface testing will focus on the way that data are entered (whether medical/data capture hierarchies and flow of data capture are logical), and the usability of the interfaces to capture data. A mixed-methods research evaluation and tools will be utilized. Data capture tools will include think aloud sessions and focus group discussions, and usability survey tools.

The DDSA prototype’s destination decisions will be tested against pre-existing decisions made by SAMU (utilizing existing SAMU databases detailing patients and destinations) and by clinical experts; iterations of the DDSA will be developed to accommodate identified errors. Iterations will be continued until decisions made by the DDSA are 90% accurate to historical and expert destination decisions. Agreed thresholds to judge usability/safety/success of the DDSA and its interfaces from discussions will be described and applied in the analysis.

#### Objective 2: Develop training materials and conduct staff training and testing in a classroom setting.

**Figure 5:**
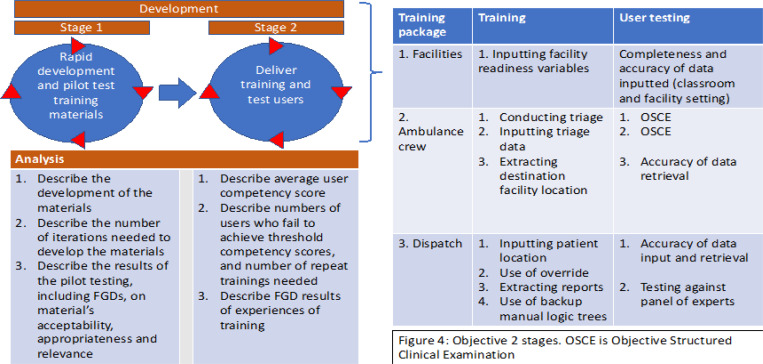
Objective 2 - stages and analysis

The second objective will proceed in two stages: (2.1) rapid development of training materials, and (2.2) delivery of training and user testing (figure 5). Training and testing materials will be developed after interfaces have reached their prefinal stage. The training materials will consist of a training booklet (including Standard Operating Procedures for each user interface), lecture materials, and quick guidelines. These materials will be piloted with users of each interface (dispatch, ambulance, health facility) using mock scenarios, with iterations of the materials developed to address issues discovered during focus group discussions with users. See appendix 4 for details.

#### Objective 2 Analyses

Figure 5 describes the main analyses that will be done for each stage of the development in objective 2. A computer simulation program for training and testing using mock scenarios for ambulance crews will be developed. Subsequently, training will be delivered, and user competency assessed with all users until target competency of ≥90% on completeness and accuracy of data inputted to their relevant interface is achieved. We will run objective structured clinical examination (OSCE) for SAMU ambulance crews for inputting triage data and for facility staff inputting readiness data. Appendix 4 describes the methodologies for objective 2 in full.

#### Iterative development

The first and second objectives are conceived to be iterative, with stages/cycles of development, testing, approvals repeating until the interfaces and DDSA are considered acceptable at pre-determined thresholds.

#### Objectives 3: Conduct mock field-trials and rollout the intervention.

The third objective tests the entire product and will proceed in three stages: (3.1) sand-box scenario testing, (3.2) mock trauma scenarios, and (3.3) rollout.

In the stage 3.1, the usage of the complete active platform will be tested by all users in classroom/computer-based simulated scenarios based on data from real patients from the Rwanda trauma registry and SAMU historical data. For this, the system will be operational in a test-server/“sandbox”. Approximately 200 scenarios will be tested over one month to ensure that all users have a chance to experience the complete system. To test each scenario will require simultaneous testing with members from facilities, dispatch, and ambulance crews.

In the stage 3.2, mock trauma scenarios will be based on real patients from the trauma registry (and/or primary trauma care guidelines) and use dummy patients (actors) at different locations in Kigali. This stage’s aims are two-fold: to test user competency and to test software reliability/functioning in the “real world”. All system users participating in the scenarios will enter and extract information in as close an approximation to the real world as possible. However, ambulance crews will not remove the “patient” from the scene, and “ambulances” may be replaced with taxis. Observers will be stationed at each “patient”, in each “ambulance”, in dispatch, and in facilities to record observations on user interaction with the software and the times that data are entered/received/extracted. Scenarios will run over 1–2 weeks and aim to involve all individual users of the system.

After these assessments are judged successful, as assessed by user competency and system reliability testing, the phase two software will be fully rolled out (stage 3.3). Roll out will be done over one month, with the current system being replaced with the new system and all study investigators being on hand to discuss and resolve any problems. All issues and solutions will be recorded and reported. Of note, roll out in Kigali will precede roll out in Musanze. Appendix 5 describes the methodologies for objective 3 in full.

## Discussion

This health system strengthening project aims to develop, test, and integrate a unique, locally developed DDSA into a bespoke emergency medical services communication system based on specific features, needs and circumstances of the Rwandan pre-hospital care system. A collaborative approach informed by the leadership of MOH/RBC, and local experts through deep engagement of key stakeholders will be utilized. The project holds great promise for improving the efficiency of emergency medical services and ultimately saving lives. To achieve this, we will work to embed 912Rwanda into policy by close engagement with all relevant stakeholder groups. Additionally, we will collaborate with multilateral policymakers from study inception to share our results and promote global uptake. Patient and public participation is a crucial aspect of our study, and our Community Engagement and Involvement (CEI) strategy ensures active involvement throughout the project—from design to dissemination. Our goal is to foster sustainable engagement in translating research into effective policies.

The capture of the development process using rigorous methodologies will produce findings which are likely to improve ambulance software development processes, including increased efficiency and quality of software products, in similar LMICs settings. That the project involves multisectoral collaboration between academia and industry will foster a deep understanding of practical challenges and facilitate the integration of theoretical and practical knowledge.

Our study has limitations, the main of which is the likely lack of availability of high-quality data in Rwanda to understand the current system and facilitate the development of the DDSA. While both SAMU and individual healthcare facilities currently collect numerous data points on emergency cases, the completeness and quality of these data may be insufficient to support a data-based approach. We have therefore developed our methods throughout the programme to use as a common foundation and situated expert practitioner opinion to mitigate against data that may be insufficient, incomplete or unavailable. Expert opinion approaches rely on input from providers in a low resourced setting and we recognize that their capacity to participate may be challenged. Nevertheless, from our experience of Phase 1, there is broad enthusiasm from collaborators across multiple sectors of the pre-hospital care system in Rwanda for this solution; together with a willingness to provide the time and resource to ensure it is developed with a high degree of fidelity and integrity for, and with the local context. Finally, we acknowledge that the field of software development is dynamic, with rapidly evolving technologies, particularly in the machine leaning space. As a result, research project and findings may become outdated relatively fast, making it essential to stay updated with the latest developments.

## Supplementary Material

Supplement 1

## APPENDIX

### Rwanda912 RIGHT Group*

#### Study leadership.

Justine Davies^1,2,3^, Jean Claude Byiringiro^4^

#### Study design.

Justine Davies^1,2,3^, Jean Claude Byiringiro^4^, Abebe Bekele^5^, Lucia D’Ambruoso^6,12,13,14,15^, Agnieszka Ignatowicz^1^, Jeanne d’Arc Nyinawankusi^7^, Jean Marie Uwitonze^7^, Jean Nepomuscene Sindikubwabo^7^, Irene Bagahirwa^7^, Collins Inkotanyi^5^, Sudha Jayaraman^8^, Antonio Belli^9^, Rob Rickard^10^.

#### Manuscript writing

Aurore Nishimwe^1*^ (first author), Assuman Nuhu^4^, Justine Davies^1,2,3^, Jean Claude Byiringiro^4^

#### Commenting on manuscript

Aurore Nishimwe^1*^, Assuman Nuhu^4^, Justine Davies^1,2,3^, Jean Claude Byiringiro^4^, Barnabas Tobi Alayande^5^, Abebe Bekele^5^, Agnieszka Ignatowicz^1^, Laura Quinn^1^, Ephrem Daniel Sheferaw^5^, Collins Inkotanyi^5^, Lucia D’Ambruoso^6,12,13,14,15^, Jeanne d’Arc Nyinawankusi^7^, Jean Marie Uwitonze^7^, Jean Nepomuscene Sindikubwabo^7^, Irene Bagahirwa^7^, Gilbert Rukundo^7^, Sudha Jayaraman^8^, Oda Munyura^5^, Fabien Hagenimana^4^, Didier Hagumimana^4^, Philbert Muhire^11^

#### Manuscript approval

All authors critically reviewed, provided feedback, and approved the final manuscript.

#### Authors affiliations

^1^School of Applied Health Sciences, College of Medicine and Health, University of Birmingham, Birmingham, UK.

^2^Centre for Global Surgery, Department of Global Health, Stellenbosch University, Cape Town, South Africa.

^3^Medical Research Council/Wits University Rural Public Health and Health Transitions Research Unit, Faculty of Health Sciences, School of Public Health, University of the Witwatersrand, Johannesburg, South Africa.

^4^College of Medicine and Health Sciences, University of Rwanda, Kigali, Rwanda.

^5^ University of Global Health Equity, Kigali, Rwanda.

^6^Aberdeen Centre for Health Data Science, Institute of Applied Health Sciences, School of Medicine, Medical Sciences and Nutrition, University of Aberdeen, Scotland, UK

^7^Rwanda Biomedical Center, Ministry of Health, Kigali, Rwanda.

^8^Department of Surgery, Center for Global Surgery, University of Utah, Salt Lake City, Utah

^9^College of Medicine and Dental Sciences, NIHR Surgical Reconstruction and Microbiology Research Centre, University of Birmingham, Birmingham, UK

^10^ Rwanda Build Program, Kigali, Rwanda.

^11^Ruhengeri Level Two Teaching Hospital, Kigali, Rwanda

^12^MRC/Wits Rural Public Health and Health Transitions Research Unit (Agincourt), School of Public Health, University of the Witwatersrand, Johannesburg, South Africa

^13^Department of Epidemiology and Global Health, Umeå University, Sweden

^14^Department of Global Health, Faculty of Medicine and Health Sciences, Stellenbosch University, South Africa

^15^Public Health, National Health Service (NHS) Grampian, Scotland, United Kingdom,

* **Corresponding author**: Aurore Nishimwe: a.nishimwe.1@bham.ac.uk
