# Supplementary material for: Developing and integrating a destination decision support algorithm into an innovative electronic communication platform to improve injury care service coordination in Rwanda: The Rwanda912 study protocol
Source: Res Sq. 2024 Dec 24:rs.3.rs-5640378. Preprint. [Version 1] doi: 10.21203/rs.3.rs-5640378/v1 (PMC11703329; doi:10.21203/rs.3.rs-5640378/v1)
Supplement: Supplement 1 [file NIHPPRS5640378V1-supplement-1.pdf]

Supplementary Files

This is a list of supplementary files associated with this preprint. Click to download.

- SupplementarymaterialIAppendix1TheoryofChange.pdf
- SupplementarymaterialIIAppendix2GanttChart.pdf
- SupplementarymaterialIIIDetailedmethodologiesAppendices36.docx
